# Association of Birth Weight with Central and Peripheral Corneal Thickness in Adulthood—Results from the Population-Based German Gutenberg Health Study

**DOI:** 10.3390/children8111006

**Published:** 2021-11-04

**Authors:** Achim Fieß, Michael S. Urschitz, Susanne Marx-Groß, Markus Nagler, Philipp S. Wild, Thomas Münzel, Manfred E. Beutel, Karl J. Lackner, Norbert Pfeiffer, Alexander K. Schuster

**Affiliations:** 1 Department of Ophthalmology, University Medical Center of the Johannes Gutenberg University Mainz, 55131 Mainz, Germany; Susanne.Marx-Gross@unimedizin-mainz.de (S.M.-G.); norbert.pfeiffer@unimedizin-mainz.de (N.P.); alexander.schuster@unimedizin-mainz.de (A.K.S.); 2 Division of Pediatric Epidemiology, Institute for Medical Biostatistics, Epidemiology and Informatics, University Medical Center of the Johannes Gutenberg University Mainz, 55131 Mainz, Germany; urschitz@uni-mainz.de; 3 Preventive Cardiology and Preventive Medicine, Center for Cardiology, University Medical Center of the Johannes Gutenberg University Mainz, 55131 Mainz, Germany; Markus.Nagler@unimedizin-mainz.de (M.N.); philipp.wild@unimedizin-mainz.de (P.S.W.); 4 Center for Thrombosis and Hemostasis (CTH), University Medical Center of the Johannes Gutenberg University Mainz, 55131 Mainz, Germany; 5 German Center for Cardiovascular Research (DZHK), Partner Site Rhine-Main, 55131 Mainz, Germany; 6 Center for Cardiology—Cardiology I, University Medical Center of the Johannes Gutenberg University Mainz, 55131 Mainz, Germany; tmuenzel@uni-mainz.de; 7 Department of Psychosomatic Medicine and Psychotherapy, University Medical Center of the Johannes Gutenberg University Mainz, 55131 Mainz, Germany; manfred.beutel@unimedizin-mainz.de; 8 Institute of Clinical Chemistry and Laboratory Medicine, University Medical Center of the Johannes Gutenberg University Mainz, 55131 Mainz, Germany; karl.lackner@unimedizin-mainz.de

**Keywords:** birth weight, cornea, corneal thickness, anatomy, epidemiology

## Abstract

**Purpose:** Low birth weight (BW) is associated with altered ocular geometry such as a steeper corneal shape in adulthood. However, it is unclear whether low birth weight affects corneal thickness development in the center or periphery in adulthood which may contribute to ocular disease. The purpose of this study was to investigate corneal thickness in former low birth weight individuals in adulthood. **Methods:** The German Gutenberg Health Study is a prospective, population-based study in which every participant (age range 40–80 years) was measured with Scheimpflug imaging (Pentacam HR, Oculus Optikgeräte GmbH, Wetzlar, Germany). BW was collected by self-reports. The relationship between birth weight and corneal thickness at different locations were assessed. Linear regression models were carried out including uni- and multivariable analyses with adjustment for age, sex, mean corneal radius, and white-to-white distance. Main outcome measures were corneal thickness at the apex, at the pupil center, and at the corneal periphery. **Results:** Overall, 5657 participants were successfully measured (3019 females, aged 56.0 ± 10.3 years). In multivariable analyses a lower BW was associated with a thinner corneal thickness at the apex (B = 1.71 µm/500 g, *p* < 0.001) and at the pupil (B = 1.69 µm/500 g, *p* < 0.001). These effects diminished towards the corneal periphery resulting in no differences in the perilimbal regions. **Conclusion:** The present study provides evidence that lower birth weight goes along with corneal thickness alterations even into adult ages of 40 to 80 years. Thinner measurements of the cornea were particularly found in the corneal center and diminished in the periphery. This indicates that there may be fetal origins affecting corneal thickness development particularly in the corneal center.

## 1. Introduction

Low birth weight (LBW) is a parameter indicating fetal growth restriction and preterm delivery. LBW and prematurity are both associated with altered ocular morphologic development leading to several unique ocular sequelae in childhood. Former preterm LBW children have altered postnatal corneal thickness [1,2,3] and increased corneal steepness [4,5,6,7,8], smaller anterior chamber depth [4,6], increased lens thickness [4], and smaller axial length [5,8]. Especially anterior segment alterations may persist until adulthood. In a recent report of the Gutenberg Health Study the authors could demonstrate that LBW affects ocular geometry and leads to a 5.5 µm thinner central cornea in individuals with LBW using optical biometry [9]. However, only central corneal thickness (CCT) was measured in this approach and the association between corneal thickness and BW towards the periphery could not be taken into account. Alterations of corneal thickness in different corneal regions might be of clinical importance in adulthood as a thinner cornea may be a first sign of corneal ectatic diseases such as keratoconus.

Overall, scarce data exist about the mechanisms influencing corneal thickness. There are few reports analyzing corneal thickness in former preterm LBW children with or without Retinopathy of Prematurity (ROP) while the long-term effects up to adulthood are hardly understood. Different studies observed an increased CCT in preterm newborns compared to full-term newborns [1,2,3,10], followed by a longitudinal thinning [11]. Others detected that birth weight (BW) is negatively correlated with CCT [12,13]. Furthermore, ROP was found as additional factor contributing to an increased CCT in newborns independent of prematurity [3]. Few studies exist analyzing the association of BW with corneal thickness in childhood [14] and adolescence [15,16]. However, some of these previous reports are limited because corneal thickness was measured with ultrasound and not with Scheimpflug imaging which is a new technology enabling corneal tomography and thickness measurements with high reliability and validity including measurements of corneal thickness at different areas of the cornea [17,18]. Using Scheimpflug imaging, the Wiesbaden Prematurity Study [5] (participants aged 4 to 10 years) and the study by Ecsedy and colleagues (participants aged 7 to 14 years) [19] found no difference for CCT between preterm and full-term children while Marques et al. [20] (participants aged 9 to 17 years) observed thinner corneas in former premature ROP individuals compared to preterms without ROP. Still, the long-term effects of LBW on corneal development is unclear.

Various factors are reported to be associated with CCT in adults such as age, gender, race, environmental, and genetic factors [21]. However, up-to-date there are no data available investigating corneal thickness and its regional distribution namely in corneal apex, pupil center, and corneal periphery in relation to BW in a population-based setting of adults using Scheimpflug imaging.

Hence, the aim of this investigation was to analyze corneal thickness and its relation to BW in different corneal regions with Scheimpflug imaging in adult individuals.

## 2. Materials and Methods

### 2.1. Study Population

The Gutenberg Health Study (GHS) is an interdisciplinary, prospective, population-based cohort study in the Rhine-Main region of Western Germany (Rhineland-Palatinate) [22]. Recruitment and baseline examination were performed between 2007 and 2012 of participants aged between 35 and 74 years. After baseline examination consecutive follow-up examinations were conducted every 5 years. All GHS participants were randomly selected from local governmental registry offices stratified by sex, age, and residence (urban or rural). Every resident has the duty to be registered in the governmental registry. Effective recruitment efficacy proportion was 55.5%. For our analysis, subjects of the 5-year follow-up performed between 2012 and 2017 were included because at this timepoint participants underwent Scheimpflug imaging.

In total, 12,423 of the original 15,010 GHS baseline participants (82.8%) took part at the 5-year follow-up examination. Written informed consent was obtained from all study participants prior to their study entry and the GHS complies with Good Clinical Practice (GCP), Good Epidemiological Practice (GEP), and the ethical principles of the Declaration of Helsinki. The study protocol and study documents were approved by the local ethics committee of the Medical Chamber of Rhineland-Palatinate, Germany (reference no. 837.020.07; original vote: 22.3.2007, latest update: 20.10.2015).

### 2.2. Birth Weight

For the present analysis, only participants with available self-reported BW were included. The participants were asked upon invitation to the GHS study center to review their records or family albums for a documented BW and were divided into the following BW groups: a LBW group < 2500 g (group 1); a normal BW group with a BW between 2500 and 4000 g (group 2); and a high BW group > 4000 g (group 3) as reported earlier [9,23,24,25]. Additionally, participants with birth weights below 1000 g and above 6000 g were excluded as these self-reported data are suspected to be unreliable. Self-reported BW distribution of the GHS participants was compared to governmental data and the medical literature as reported earlier [24,26].

### 2.3. Ophthalmologic Examination

Detailed ophthalmologic examination was performed in every study participant as described earlier [27]. These included testing of visual acuity and objective refraction (Humphrey^®^ Automated refractor/Keratometer (HARK) 599™), and intraocular pressure measurement with a noncontact tonometer (NT 2000™, Nidek Co./Tokio, Japan). Scheimpflug imaging (Pentacam HR™, Oculus, Wetzlar, Germany) and optical biometry (Lenstar LS900, Haag-Streit, Bern, Switzerland) was additionally conducted [27]. The spherical equivalent was calculated by adding the spherical correction value to half the cylinder value.

### 2.4. Scheimpflug Imaging

Corneal and anterior segment tomography was conducted in every participant using a rotating Scheimpflug camera. This device allows a three-dimensional examination from the anterior corneal surface to the posterior lens surface. Strict SOPs were used for performing Scheimpflug imaging to reduce examiner depending variance. In the case of optimal alignment corneal tomography was started while participants had to fixate a light source. During the Scheimpflug examination, 25 Scheimpflug images are captured in about 2 s. Quality controls of the device were checked. All corneal thickness measurements were controlled for outliers and outliers were removed if a measurement artifact was suspected based on the raw Scheimpflug images. The following corneal parameters were included in the present analysis: central corneal thickness in the pupil center, corneal thickness at the apex, corneal center (thinnest corneal thickness) (D0) and circles around the thinnest corneal position with 2 mm (D2), 4 mm (D4), 6 mm (D6), 8 mm (D8), and 10 mm (D10) diameter.

### 2.5. Covariates

The following factors previously reported to be associated with corneal thickness [21,28,29] were considered. This included: (1) age [29]; (2) gender [28]; (3) mean corneal radius [21]; (4) white-to-white distance [21].

### 2.6. Statistical Analysis

Linear regression models with general estimating equations (GEE) were used to assess associations and to account for correlations between corresponding eyes. If available both eyes were included into the analyses. In model #1, the relationship between BW as independent variable and the main outcome measures was investigated in a crude model; in model #2, the association was adjusted for sex, age, mean corneal radius, and white-to-white distance. All models were computed with BW as a continuous variable and as a categorical variable (i.e., BW < 2500 g, BW between 2500 and 4000 g, BW > 4000 g). The data were analyzed with R version 3.6.1 [30].

This is an explorative study and no adjustment for multiple testing was carried out. Thus, *p* values should be regarded as a continuous parameter reflecting the level of confidence; and are therefore reported exactly. A *p*-value <0.001 was considered as strong association, *p* < 0.05 as likely association, *p* > 0.05 but <0.6 as inconclusive and *p* ≥ 0.6 as probably not associated.

## 3. Results

### 3.1. Participant Characteristics

Of 15,010 subjects examined at baseline 12,423 returned for the 5-year follow-up examination of which 7194 participants reported their BW between 1000 and 6000 g. Furthermore, 1010 were excluded because of missing Scheimpflug examination and 121 because of a history or missing data of corneal surgery. In total, 406 were excluded because of self-reported eye injury or missing data about eye injury. Overall, 5657 participants were included into the final analysis. Characteristics such as age, sex, refractive error, and corneal curvature are presented in Table 1. Mean age at examination was 56.0 ± 10.3 years and 53.4% (*n* = 3019) were female. A BW below 2500 g (group 1) was documented by 315 participants, BW between 2500 g and 4000 g (group 2) was given by 4655 participants, and BW above 4000 g (group 3) was provided by 687 participants. In Table 2 measurements of corneal thickness in different corneal areas stratified by BW groups are displayed.

#### Non-Responder Analysis

Participants with documented self-reports of BW and Scheimpflug measurement were younger (56.0 ± 10.3 years versus 62.4 ± 10.5 years) and more likely to be female (53.4% versus 45.0%) compared to excluded participants (Supplementary Table S1).

### 3.2. Birth Weight as a Continuous Variable

#### 3.2.1. Corneal Thickness at the Corneal Apex and Pupil

**Figure 1:**
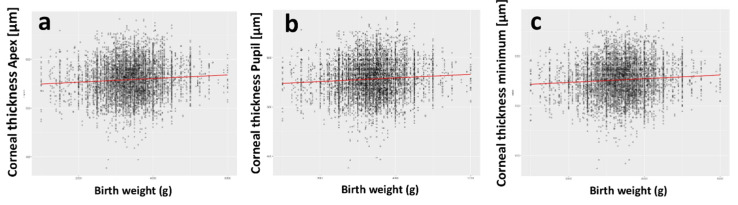
Scatterplot of birth weight with (**a**) corneal thickness in the apex, (**b**) corneal thickness in the pupil center, (**c**) minimal corneal thickness in the Gutenberg Health Study (*n* = 5657).

**Figure 2:**
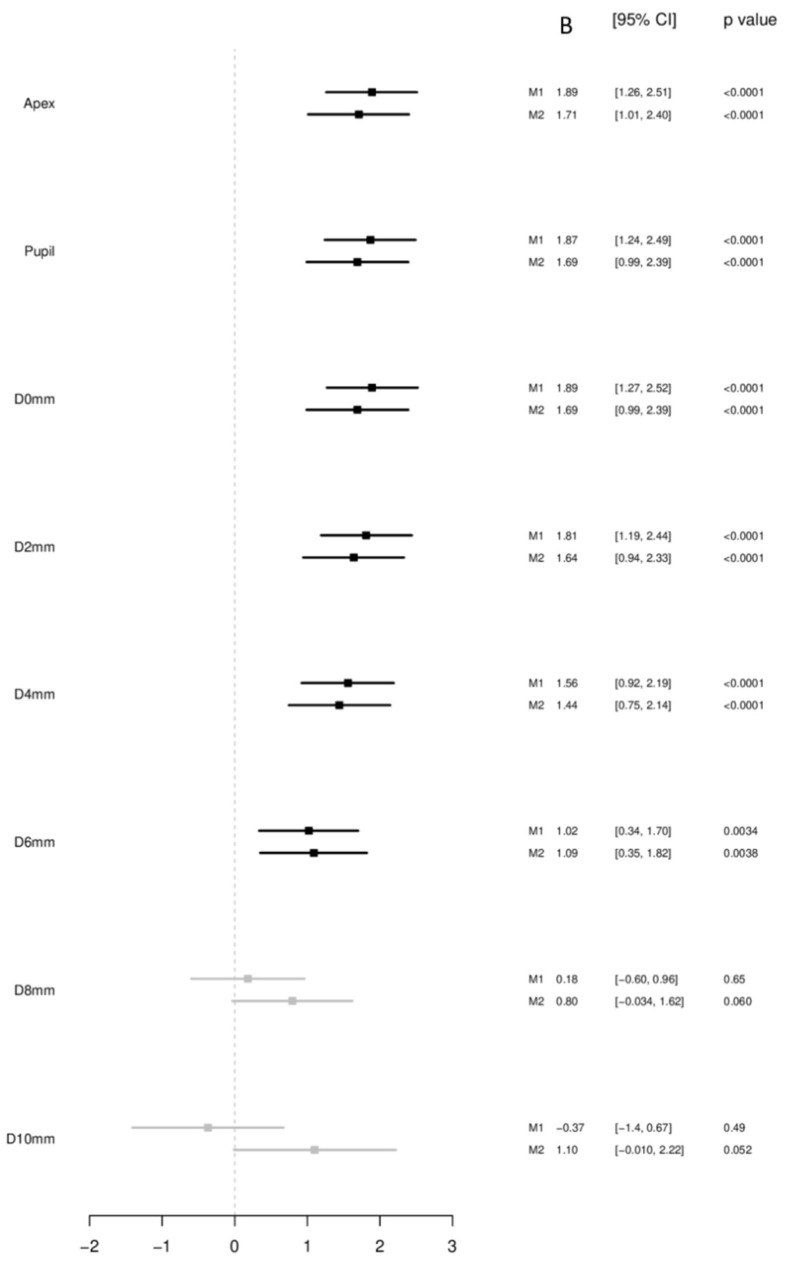
Associations of corneal thickness in the different corneal regions with birth weight (continuous; *n* = 5657) in the Gutenberg Health Study (2012–2017). B—Beta; CI—Confidence interval. Linear regression analysis using generalized estimating equations to control for correlations between right and left eyes. Estimates are present per 500 g. B—Beta; CI—Confidence interval. M1 Crude model without adjustment. M2 Model adjusted for age; sex; mean corneal radius, white-to-white distance. A *p*-value <0.001 was considered as strong association, *p* < 0.05 as likely association, *p* > 0.05 but <0.6 as inconclusive and *p* ≥ 0.6 as probably not associated.

A scatterplot of BW with corneal thickness in the apex, corneal thickness in the pupil center, and minimal corneal thickness is displayed in Figure 1. BW was associated with corneal thickness in the unadjusted analysis and in the multivariable model at the apex (per 500 g: B = 1.71 µm [95%-CI: 1.01; 2.40], *p* < 0.001) and at the pupil center (per 500 g: B = 1.69 µm [95%-CI: 0.99; 2.39], *p* < 0.001) (Figure 2).

#### 3.2.2. Corneal Thickness Minimum and Periphery

BW showed also a strong association with minimal corneal thickness (D0) in unadjusted analysis and in the multivariable model (per 500 g: B = 1.69 µm [95%-CI: 0.99; 2.39], *p* < 0.001).

There was a gradient of association between BW and corneal thickness with respect to measurement position: the more central the measurement was the stronger the association. In circles around corneal thickness minimum with diameters of 2 mm (per 500 g: B = 1.64 µm [95%-CI: 0.94; 2.33], *p* < 0.001); 4 mm (per 500 g: B = 1.44 µm [95%-CI: 0.75; 2.14], *p* = 0.004); 6 mm (per 500 g: B = 1.09 µm [95%-CI: 0.35; 1.82], *p* = 0.004) associations were found, while with 8 mm (per 500 g: B = 0.80 µm [95%-CI: −0.034; 1.62], *p* = 0.060) as well as with 10 mm (per 500 g: B = 1.10 µm [95%-CI: −0.01; 2.22], *p* = 0.052), inconclusive associations were observed (Figure 2).

### 3.3. Birth Weight Categorized into Low, Normal and High Birth Weight

#### 3.3.1. Corneal Thickness at the Corneal Apex and Pupil

When analyzing associations with BW groups, the LBW group showed in multivariable analyses, a likely association with smaller corneal thickness at the apex (<2500 g: B = −4.4 µm [95%-CI: −8.3; −0.51], *p* = 0.026) and at the pupil center (<2500 g: B = −4.2 µm [95%-CI: −8.1; −0.30], *p* = 0.035) compared to the BW reference group.

**Figure 3:**
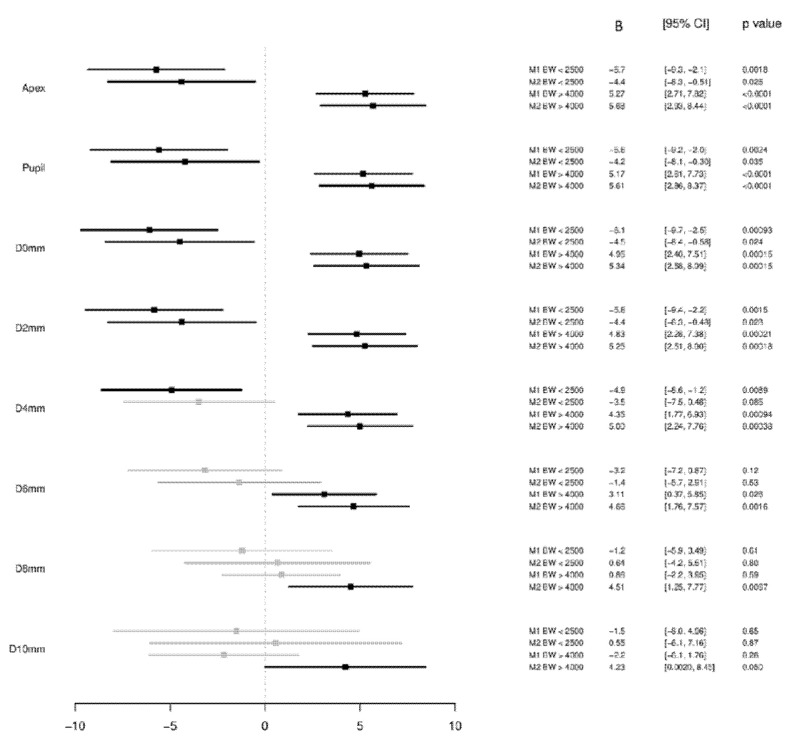
Associations of corneal thickness in the different corneal regions with Birth Weight Groups (low birth weight *n* = 315; normal birth weight *n* = 4655; high birth weight *n* = 687) in the Gutenberg Health Study (2012–2017). B—Beta; CI—Confidence interval. Linear regression analysis using generalized estimating equations to control for correlations between right and left eyes. Estimates are present per 500 g. B—Beta; CI—Confidence interval. M1 Crude model without adjustment. M2 Model adjusted for age; sex; mean corneal radius, white-to-white distance. A *p*-value < 0.001 was considered as strong association, *p* < 0.05 as likely association, *p* > 0.05 but <0.6 as inconclusive and *p* ≥ 0.6 as probably not associated.

Individuals with higher BW showed a strong association with higher corneal thickness at the apex (>4000 g: B = 5.68 µm [95%-CI: 2.93; 8.44], *p* < 0.001) and at the pupil center (>4000 g: B = 5.61 µm [95%-CI: 2.86; 8.37], *p* < 0.001) compared to the BW reference group (Figure 3).

#### 3.3.2. Corneal Thickness Minimum and Periphery

The minimal corneal thickness was likely associated with LBW when compared to the BW reference group (<2500 g: B = −4.5 µm [95%-CI: −8.4; −0.58], *p* = 0.024) as it was observed for the distance at 2 mm (<2500 g: B = −4.4 µm [95%-CI: 8.3; −0.48], *p* = 0.028). No conclusive association was observed for the distance at 4 mm (<2500 g: B = −3.5 µm [95%-CI: −7.5; 0.48], *p* = 0.085) and 6 mm (<2500 g: B = −1.4 µm [95%-CI: −5.7; 2.91], *p* = 0.53), while probably no association at 8 mm (<2500 g: B = 0.64 µm [95%-CI: −4.2; 5.51], *p* = 0.80) and at 10 mm distance (<2500 g: B = 0.55 µm [95%-CI: −6.1; 7.16], *p* = 0.87).

Minimal corneal thickness was strongly associated with high BW when compared to the BW reference group (>4000 g: B = 5.34 µm [95%-CI: 2.58; 8.09], *p* < 0.001) as it could be detected for the distance at 2 mm (>4000 g: B = 5.25 µm [95%-CI: 2.51; 8.00], *p* < 0.001), at 4 mm (>4000 g: B = 5.00 µm [95%-CI: 2.24; 7.76], *p* < 0.001), at 6 mm (>4000 g: B = 4.66 µm [95%-CI: 1.76; 7.57], *p* = 0.002) and a likely association at 8 mm (>4000 g: B = 4.51 µm [95%-CI: 1.25; 7.77], *p* = 0.007) and an inconclusive association at 10 mm (>4000 g: B = 4.23 µm [95%-CI: 0.002; 8.45], *p* = 0.050) distance (Figure 3).

## 4. Discussion

This study presents new population-based results about the relationship of BW and corneal thickness and its spatial distribution using Scheimpflug imaging in a large cohort of adults. Former LBW individuals have thinner corneas at the apex and at the pupil, while these differences diminished towards the periphery. Furthermore, former high BW adults revealed an increased corneal thickness compared to normal BW individuals. Our data give new insights into the long-term effects of lower BW on corneal thickness indicating that there are most likely fetal origins of corneal morphology development particularly in the corneal center with less effects on corneal periphery.

These findings may be of clinical importance as a thinner cornea may be a risk factor for ocular diseases such as keratoconus which is an ectatic corneal disorder associated—amongst others—with corneal thinning. In the last decades, only few studies investigated the etiology of keratoconus and few risk factors including eye rubbing, parents’ education (as a measure of socio-economic status) and genetic markers were observed [31]. This only explains partially keratoconus occurrence while unknown risk factors are suspected and our new results might be a first hint for a potential association as we found a thinner corneal apex and corneal minimum.

Our study extents literature which has mainly investigated the effects of extreme prematurity, extreme LBW and postnatal ROP occurrence or treatment on CCT development in infancy and childhood. A review of the literature revealed reports of thicker corneas in preterm LBW newborns [1,2,3,10] and a decrease of CCT until premature infants reach full-term age [10,32,33]. This decrease might be modulated by ROP occurrence: in a recent report of Kardaras et al. [3] the authors examined preterm newborns (gestational age between 24 and 35 weeks) with and without ROP in 1- or 2-week intervals between 30 and 38 weeks of postmenstrual age. The authors found that CCT decrease is lower in preterm infants with ROP (10.93 µm per week) than in preterm infants without ROP (16.11 µm/week) resulting in different final CCT values at a postmenstruational age of 38 weeks (571.52 ± 37.05 µm with ROP versus 541.85 ± 77.56 µm without ROP), which is comparable to other previous reports [1,11,34]. Others detected that BW is negatively correlated with CCT [12,13] and Acar et al. observed a longitudinal thinning after preterm birth [11]. Portellinha and Belfort [35] examined central and peripheral corneal thickness in newborns and observed that full-term newborns with 2.500 to 3.000 g had a higher peripheral corneal thickness compared to newborns with 3.501 to 4.000 g. In contrast, Remón et al. [32] found in 1- to 6-days-old newborns no statistical correlation between corneal thickness and BW. Some investigators hypothesized that the difference of CCT diminishes in preterm infants until they reach full-term age [35,36]. Yeter et al. observed that all regions of corneal thicknesses (with the exception of pericentral corneal thickness) were correlated with BW in children of 3–6 years of age [14] whereas Fledelius et el. [16] reported a permanent effect of low BW on the cornea investigating former preterm and full-term individuals at the ages of 10 and 18 years. In congruence, a 19.35µm thinner central cornea was observed in Chinese adolescents that had LBW compared to former normal BW subjects using optical biometry [15]. Previous data from the Gutenberg Health Study showed that former LBW adults had a 5 µm thinner central corneal thickness compared to normal BW individuals using optical biometry [9]. The present study extents these previous findings and presents new population-based results that LBW adults have thinner corneas at the apex, at the pupil center and at its minimum, while these differences diminish towards the periphery.

Using Scheimpflug imaging, there are three studies investigating corneal thickness in premature children. The Wiesbaden Prematurity Study observed no difference between former preterm infants with and without ROP compared to former full-term infants in the age of 4 to 10 years [5]. This is congruent to data of Ecsedy et al. who analyzed data of former preterm children (535 ± 103 µm) versus former full-term children (561 ± 33 µm; *p* = 0.09) aged 7 to 14 years [19]. Marques investigated 39 premature patients without ROP and 39 eyes with a history of ROP (aged 9 to 17 years) and report a significant thinner cornea in eyes with history of ROP [20].

We can only speculate about the long-term mechanisms in LBW individuals contributing to a thinner cornea. Possible explanations might be structural changes due to altered corneal remodeling processes and potential ultrastructural changes in collagen fiber layers. Furthermore, as an explanatory model, it is hypothesized that increased intrauterine CCT is caused by a longer duration of closed eyes while the decrease of corneal thickness after birth is caused by improved corneal hydration, evaporation and corneal remodeling processes and an elongation of collagen fibers elements [1,36]. Furthermore, Fielder and colleagues speculated that lower extrauterine environmental temperature after preterm birth leads to altered corneal geometry [37]. This might be another unknown factor potentially contributing to altered long-term corneal thickness development. As gestational age and ROP were not documented within our study we cannot investigate these factors. However, it is noteworthy that these processes seem to be particularly present at the corneal center.

Several reports exist comparing CCT in different populations [28,38,39,40] as well as analyzing associations with general parameters. Dai and Gunderson [41] found that CCT varied among different ethnic groups in a pediatric population, African-Americans had thinner corneas than Caucasians and Hispanics. With respect to sex, men had a thicker CCT [42,43], and studies reported an age-related decrease of CCT [44,45]. Furthermore, CCT is negatively associated with body height [46] and positively associated with body weight in men and with body mass index in all subjects [40]. These parameters are known to be—at least partially—influenced by LBW and LBW may to some extent explain those associations with altered corneal thickness [5,7,9] and ocular geometry [47,48,49,50,51,52].

### 4.1. Strengths and Limitations

The major limitation of the present analysis is that around 44% of the participants did not provide self-reported BW data and these data could not be validated by individual chart review of medical records. Thus, we are not able to exclude or cope with misclassification. The purpose of the request to each study participant were asked to review personal and familiar birth documents to ensure a high validity of self-reported BW data. In the Australian Twin Study, a high intraclass correlation for self-reported BW data was demonstrated [53]. Furthermore, the distribution of self-reported BW data were compared to data from the German Federal Statistical Office of the early 1970s, which revealed comparable distributions of low, normal and high BW groups [24,26]. As a result, we expect that our results can be generalized to the German population.

Another important limitation is the lack of data about gestational age and postnatal ROP occurrence and treatment. Previous studies focused on former extreme LBW individuals [9] while we analyzed the relationship in a population-based sample. Thus, we were not able to investigate the effects of extreme prematurity and associated comorbid factors such as ROP in our study. Furthermore, there is the possibility that even moderate prematurity could have an impact on the development of the cornea and it should be considered that missing gestational age might be a confounding factor in our association analyses. In addition, we cannot make a conclusion about the effects of perinatal nutrition status in correlation to gestational age, for instance being small or large for gestation age.

Accuracy of corneal thickness measurements can vary and depend on the device used. Within our study we used non-contact Scheimpflug technology for corneal thickness measurement, which was evaluated in several studies showing high reproducibility and repeatability [54,55] when compared to ultrasound biometry.

The major strengths of our study are the population-based sample and the large number of participants. Every investigator was blinded to BW data to avoid information bias and each examination was strictly performed according to standard operation procedures.

### 4.2. Summary

We presented new results about potential long-term effects of low BW on corneal thickness in adulthood. We demonstrated that corneal thickness at the apex, at the pupil center, and at its minimum was thinner in former low BW adults. Thinner measurements of the cornea were particularly found in the corneal center and diminished in the periphery. These results indicate that adult corneal thickness morphology may have origins—amongst others—in early life. The thinner central cornea may predispose individuals to corneal diseases, i.e., ectatic corneal diseases.

## Figures and Tables

**Table 1:** Characteristics of the study sample (*n* = 5657). Data from the population-based Gutenberg Health Study (2012-17) by sex groups. Mean ± Standard Deviation or Median and 25%/75% Quantiles.

Variable	Overall	Male	Female
Participants (*n*)	5657	2638	3019
Sex (Women)	53.4% (3019)	0% (0)	100% (3019)
Age (y)	56.0 ± 10.3	56.1 ± 10.3	56.0 ± 10.3
Height (cm)	171 ± 10	178 ± 7	164 ± 7
Weight (kg)	79.9 ± 17.2	88.7 ± 14.7	72.1 ± ^5.3
BMI	26.5 (23.7/30.0)	27.2 (24.9/30.2)	25.6 (22.7/29.7)
Socioeconomic status (score)	13.78 ± 4.26	14.55 ± 4.22	13.11 ± 4.18
Birth weight (g)	3395 ± 648	3530 ± 650	3277 ± 623
**Cardiovascular parameters**			
Hypertension (yes)	46.6% (2632)	53.1% (1401)	40.8% (1231)
Diabetes (yes)	7.4% (421)	9.7% (255)	5.5% (166)
Dyslipidemia (yes)	29.6% (1673)	37.9% (997)	22.4% (676)
Smoker (yes)	16.7% (942)	17.3% (457)	16.1% (485)
**Ocular disease:**			
Self-reported glaucoma (yes)	3.1% (176)	3.1% (77)	3.2% (99)
Self-reported AMD (yes)	1.3% (71)	1.0% (27)	1.5% (44)
**Ocular parameters:**			
Visual acuity OD (logMAR)	0.10 (0/0.20)	0 (0/0.10)	0.10 (0/0.20)
Visual acuity OS (logMAR)	0.00 (0/0.10)	0 (0/0.10)	0 (0/0.10)
Spherical equivalent OD (diopter)	−0.25 (−1.50/0.62)	−0.25 (−1.50/0.62)	−0.25 (−1.50/0.75)
Spherical equivalent OS (diopter)	−0.25 (−1.50/0.62)	−0.25 (−1.50/0.62)	−0.25 (−1.50/0.75)
Intraocular pressure OD (mmHg)	14.73 ± 2.96	14.89 ± 3.11	14.59 ± 2.81
Intraocular pressure OS (mmHg)	14.80 ± 2.95	14.99 ± 3.06	14.65 ± 2.84
Mean corneal radius OD (mm)	7.77 ± 0.28	7.84 ± 0.28	7.70 ± 0.26
Mean corneal radius OS (mm)	7.77 ± 0.28	7.84 ± 0.28	7.71 ± 0.26
White-to-white OD (mm)	12.2 ± 0.4	12.3 ± 0.4	12.1 ± 0.4
White-to-white OS (mm)	12.2 ± 0.4	12.3 ± 0.4	12.2 ± 0.4
Axial length OD (mm)	23.8 ± 1.3	24.1 ± 1.3	23.5 ± 1.3
Axial length OS (mm)	23.8 ± 1.3	24.1 ± 1.3	23.5 ± 1.2

*n*—number of participants, cm—centimeter, kg—kilogram; g—gram; mm-millimeter; y—year; AMD—age-related macular degeneration: OD—right eye; OS—left eye.

**Table 2:** Measurements of corneal thickness in different corneal areas stratified by birth weight groups with Scheimpflug tomography. Data from the population-based Gutenberg Health Study (2012-17). Mean ± Standard Deviation or Median and 25%/75% Quantiles.

Variable in µm	<2.5 kg (315)	2.5–4.0 kg (4655)	>4.0 kg (687)	*p* Value
Corneal thickness				
Apex OD	552 ± 32	558 ± 32	563 ± 32	<0.001
Apex OS	553 ± 32	558 ± 33	564 ± 32	<0.001
Pupil OD	551 ± 32	556 ± 32	561 ± 32	<0.001
Pupil OS	551 ± 32	557 ± 33	562 ± 32	<0.001
Corneal thickness in circles				
around corneal thickness minimum				
D 0 mm OD	546 ± 32	552 ± 33	557 ± 32	<0.001
D 0 mm OS	546 ± 32	552 ± 33	557 ± 33	<0.001
D 2 mm OD	555 ± 32	561 ± 32	566 ± 32	<0.001
D 2 mm OS	555 ± 32	561 ± 33	566 ± 33	<0.001
D 4 mm OD	583 ± 33	588 ± 33	592 ± 33	<0.001
D 4 mm OS	583 ± 33	588 ± 33	592 ± 33	<0.001
D 6 mm OD	627 ± 37	630 ± 35	633 ± 35	0.013
D 6 mm OS	627 ± 36	630 ± 35	633 ± 35	0.007
D 8 mm OD	690 ± 42	692 ± 40	692 ± 39	0.54
D 8 mm OS	691 ± 42	692 ± 40	693 ± 39	0.36
D 10 mm OD	776 ± 54	777 ± 51	774 ± 47	0.58
D 10 mm OS	777 ± 53	778 ± 50	776 ± 48	0.86

All corneal thickness values are reported in µm. OD—right eye; OS—left eye; D—Distance.

## Data Availability

M.N. and P.S.W. had full access to all the data in the study and take responsibility for the integrity of the data and the accuracy of the data analysis. Statistical analyses were performed by A.K.S. The analysis presents clinical data of a large-scale population-based cohort with ongoing follow-up examinations. This project constitutes a major scientific effort with high methodological standards and detailed guidelines for analysis and publication to ensure scientific analyses on highest level. Therefore, data are not made available for the scientific community outside the established and controlled workflows and algorithms. To meet the general idea of verification and reproducibility of scientific findings, we offer access to data at the local database in accordance with the ethics vote upon request at any time. The GHS steering committee, which comprises a member of each involved department and the coordinating PI of the Gutenberg Health Study (P.S.W.), convenes once a month. The steering committee decides on internal and external access of researchers and use of the data and biomaterials based on a research proposal to be supplied by the researcher. Interested researchers make their requests to the coordinating PI of the Gutenberg Health Study (Philipp S. Wild; philipp.wild@unimedizin-mainz.de). More detailed contact information is available at the homepages of the GHS (www.gutenberghealthstudy.org) (16 September 2021).

## Supplementary Materials

The following are available online at https://www.mdpi.com/article/10.3390/children8111006/s1, Table S1: Characteristics of the GHS study sample (2012-17) stratified for included and excluded participants (Mean ± Standard Deviation or Median and 25%/75% Quantiles).
