# Supplementary material for: Association of Birth Weight with Central and Peripheral Corneal Thickness in Adulthood—Results from the Population-Based German Gutenberg Health Study
Source: Children (Basel). 2021 Nov 4;8(11):1006. doi: 10.3390/children8111006 (PMC8625912; doi:10.3390/children8111006)
Supplement: Supplementary file 1 [file children-08-01006-s001.zip › children-1406232-supplementary.pdf]

Supplementary Table S1. Characteristics of the GHS study sample (2012-17) stratified for included and excluded participants (Mean ± Standard Deviation or Median and 25%/75% Quantiles).

Variable	overall	included	excluded
Participants (n)	12,423	5657	6766
Sex (Women)	48.8% (6065)	53.4% (3019)	45.0% (3046)
Age (y)	59.5 ± 10.8	56.0 ± 10.3	62.4 ± 10.5
Height (cm)	170 ± 10	171 ± 10	170 ± 9
Weight (kg)	80.1 ± 16.8	79.9 ± 17.2	80.3 ± 16.5
BMI	26.8 (24.1/30.2)	26.5 (23.7/30.0)	27.0 (24.4/30.3)
Socioeconomic status (score)	13.02 ± 4.44	13.78 ± 4.26	12.38 ± 4.48
Birth weight (g)	3408 ± 653	3395 ± 648	3456 ± 668
**Cardiovascular parameters**			
Hypertension (yes)	53.9% (6686)	46.6% (2632)	60.0% (4054)
Diabetes (yes)	10.6% (1313)	7.4% (421)	13.2% (892)
Dyslipidemia (yes)	34.6% (4291)	29.7% (1673)	38.8% (2618)
Smoker (yes)	15.1% (1875)	16.7% (942)	13.8% (933)
**Ocular parameters:**			
Visual acuity OD (logMAR)	0.10 (0/0.20)	0 (0/0.20)	0.10 (0/0.20)
Visual acuity OS (logMAR)	0.10 (0/0.20)	0 (0/0.10)	0 (0/0.20)
Spherical equivalent OD (diopter)	−0.12 (−1.25/0.88)	−0.25 (−1.50/0.62)	0.0 (−1.00/1.12)
Spherical equivalent OS (diopter)	−0.12 (−1.25/0.88)	−0.25 (−1.50/0.62)	0.0 (−1.00/1.12)
Intraocular pressure OD (mmHg)	14.75 ± 2.96	14.73 ± 2.96	14.78 ± 2.97
Intraocular pressure OS (mmHg)	14.84 ± 3.01	14.80 ± 2.95	14.88 ± 3.07
Mean corneal radius OD (mm)	7.77 ± 0.28	7.77 ± 0.28	7.76 ± 0.27
Mean corneal radius OS (mm)	7.76 ± 0.28	7.77 ± 0.28	7.75 ± 0.27
White-to-white OD (mm)	12.2 ± 0.4	12.2 ± 0.4	12.2 ± 0.4
White-to-white OS (mm)	12.2 ± 0.4	12.3 ± 0.4	12.2 ± 0.4
Axial length OD (mm)	23.7 ± 1.3	23.8 ± 1.3	23.7 ± 1.2
Axial length OS (mm)	23.7 ± 1.3	23.8 ± 1.3	23.6 ± 1.2

n—number of participants, cm—centimeter, kg—kilogram; g—gram; mm—millimeter; OD—right eye; OS—left eye.
